# Dataset on interactors of the *Arabidopsis thaliana* Plant Natriuretic Peptide (AtPNP-A) determined by mass spectrometry

**DOI:** 10.1016/j.dib.2020.105606

**Published:** 2020-04-22

**Authors:** Ilona Turek, Helen Irving, Chris Gehring

**Affiliations:** a Biomolecular Laboratory, Division of Biological and Environmental Sciences and Engineering, King Abdullah University of Science and Technology, Thuwal, Saudi Arabia; b Department of Pharmacy and Biomedical Sciences, La Trobe Institute for Molecular Science, La Trobe University, Bendigo, Australia; c Monash Institute of Pharmaceutical Sciences, Monash University, Melbourne, Australia; d Department of Chemistry, Biology & Biotechnology, University of Perugia, 06121 Perugia, Italy

**Keywords:** Plant natriuretic peptide, peptide hormone signalling, interactors of AtPNP-A, *Arabidopsis thaliana*

## Abstract

Interactors of the plant natriuretic peptide present in *Arabidopsis thaliana*, termed AtPNP-A, were affinity-based isolated from *A. thaliana* (Col-0) leaf mesophyll cell protoplasts by incubating the protoplasts with biologically active biotinylated peptide corresponding to amino acid sequence of the active site of AtPNP-A (pAtPNP-A), either in the presence or absence of a cross-linking agent, 3,3′-dithiobis(sulfosuccinimidyl propionate) (DTSSP), or with equimolar amount of biotin with DTSSP (negative control). Upon biotin/streptavidin-based isolation of proteins bound to the pAtPNP-A or biotin, the proteins were separated by sodium dodecyl sulphate – polyacrylamide gel electrophoresis (SDS-PAGE), digested with trypsin and subjected to identification with liquid chromatography tandem mass spectrometry (LC-MS/MS). Label-free quantification of identified proteins allowed identification of binding partners of AtPNP-A, paving the way for pinpointing novel signal transduction pathways AtPNP-A is involved in. The raw and processed LC-MS/MS data reported in this article have been deposited to the ProteomeXchange Consortium with the dataset identifier PXD017925.

Specifications Table

**Table:** 

Subject	Plant Science
Specific subject area	*Arabidopsis thaliana*, plant natriuretic peptide interactors, protein-protein interactions
Type of data	Table Figure
How data were acquired	Tryptic peptides obtained from SDS-PAGE separation of affinity-based isolated proteins were analysed using LTQ Orbitrap Velos mass spectrometer (Thermo Scientific)
Data format	Raw, processed and analysed MS data
Parameters for data collection	Mesophyll cell protoplasts from *A. thaliana* (Col-0) leaves were incubated with biologically active biotinylated peptide containing amino acid sequence of the active site of *A. thaliana* plant natriuretic peptide, AtPNP-A, in the presence or the absence of DTSSP. As a negative control, the protoplasts were incubated with biotin in the presence of DTSSP. Proteins isolated with streptavidin-coupled Dynabeads M-280 were separated on SDS-PAGE and digested with trypsin
Description of data collection	LC-MS/MS data were obtained using LTQ Orbitrap Velos mass spectrometer. Data were recorded with the Xcalibur software (Thermo Scientific) and converted with Proteome Discoverer (Thermo Scientific). All spectra were submitted to Mascot (Matrix Science) and searched against *A. thaliana* in the TAIR database
Data source location	Thuwal, Saudi Arabia
Data accessibility	Public repository name: ProteomeXchange Consortium Data identification number: PXD017925 Direct URL to data: http://www.ebi.ac.uk/pride/archive/projects/PXD017925 Supplementary Table 1 and additional files

## Value of the data

• Mass spectrometric data and analysis of the pattern of mesophyll cell proteins over-represented in samples containing proteins captured with biotinylated AtPNP-A peptide in the presence or absence of the cross-linking agent, relative to abundance of proteins bound to biotin, can be compared with data from other authors.
• Researchers investigating function of plant natriuretic peptides in regulating processes including water-salt balance and response to biotic and abiotic stress can benefit from these data.
• The data presented may assist in confident identification of potential binding partners of AtPNP-A, which may lead to elucidation of novel signalling pathways AtPNP-A involved in.
• The results are valuable for understanding the molecular mechanism of plant natriuretic peptides in other plants and are key for further exploration of their signalling in modulating cellular homeostasis and plant response to stresses.

## 1 Data Description

**Fig. 1:**
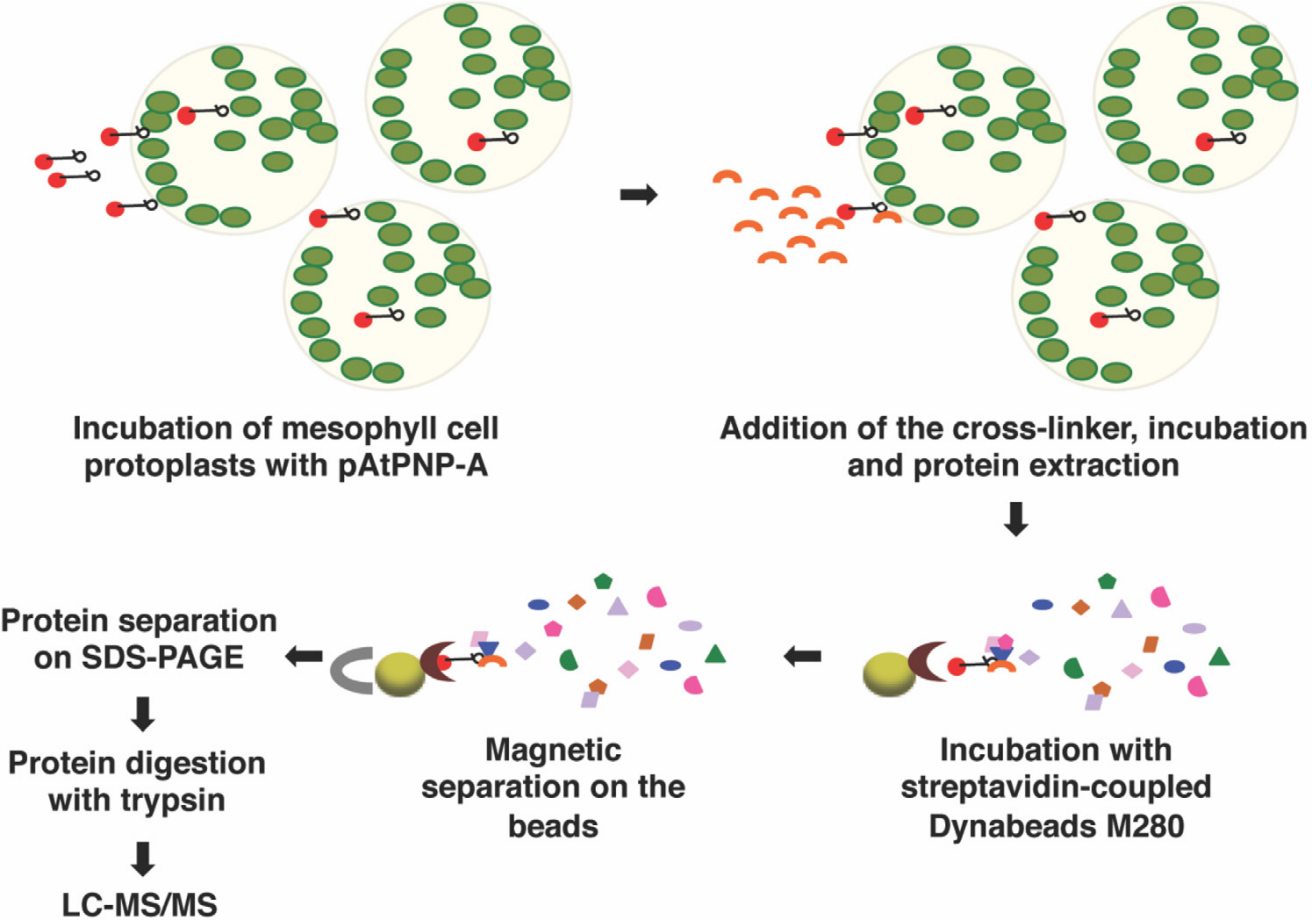
Design of the cross-linking followed by affinity-based isolation and LC-MS/MS identification of AtPNP-A interactors. Mesophyll cell protoplasts were incubated with 2.5nmol of biologically active biotinylated AtPNP-A peptide (pAtPNP-A; Genscript, USA; pAtPNP-A indicated in black and biotin by red dot) or biotin (negative control) for 20 min. Cross-linking reaction was performed with the use of DTSSP (Thermo Scientific; indicated with orange crescent) added or not to the protoplasts. After 1 h incubation on ice, the reactions were terminated and the proteins were extracted. Upon affinity-based separation of the proteins bound to biotinylated (red dot) pAtPNP-A (black) in the presence or the absence of the cross-linker, or proteins bound to biotin in the presence of the cross-linker (negative control) on the beads coupled with streptavidin (indicated with brown crescent), non-specifically bound proteins were washed off and the interactors were eluted from the beads under denaturing conditions and separated by SDS-PAGE. Proteins extracted from the excised gel bands were in-gel digested with trypsin and the resulting peptides were subjected to LC-MS/MS analysis.

This dataset comprises the acquired MS raw data (.raw files), processed ‘result’ and ‘peak’ data (.mzid, and .mgf files, respectively), and analysed data in MS Excel (.xlsx) file (Supplementary Table 1) generated from label-free pattern analysis of MS data from affinity-based isolation of interactors of AtPNP-A in *A. thaliana* (Col-0) leaf mesophyll cell protoplasts, outlined in Figure 1, with Scaffold Q+. Results of relative quantification of total spectrum counts corresponding to proteins identified with a confidence level > 99% at false discovery rate (FDR) < 0.1%, from three independent experiments are given in Table 1.

**Table 1:** Proteins predicted to interact with AtPNP-A based on affinity-based capturing followed by LC-MS/MS analyses.

Protein name	Accession Number (TAIR)	ANOVA (P <0.05)
2-oxoacid dehydrogenases acyl transferase family protein (LTA2)	AT3G25860.1	< 0.00010
Dicarboxylate diiron protein, putative (Crd1)	AT3G56940.1	< 0.00010
Ribosomal protein L14p/L23e family protein	AT1G04480.1, AT2G33370.1, AT3G04400.1, AT3G04400.2	< 0.00010
Glyceraldehyde 3-phosphate dehydrogenase A subunit 2 (GAPA-2)	AT1G12900.1, AT1G12900.3, AT1G12900.4	< 0.00010
Aldolase-type TIM barrel family protein	AT3G14415.1, AT3G14415.2, AT3G14415.3	0.00011
Ribosomal protein L3 family protein	AT2G43030.1	0.00011
ATPase, F1 complex, alpha subunit protein	AT2G07698.1	0.00024
Light harvesting complex photosystem II (LHCB4.3)	AT2G40100.1	0.00027
Aldolase-type TIM barrel family protein	AT3G14420.1, AT3G14420.2, AT3G14420.4, AT3G14420.5, AT3G14420.6	0.00031
Ribosomal protein L18e/L15 superfamily protein	AT5G27850.1	0.00041
Photosystem I light harvesting complex gene 1 (LHCA1)	AT3G54890.1, AT3G54890.4	0.00053
ATPase, F0 complex, subunit B/B', bacterial/chloroplast (ATPF)	ATCG00130.1	0.00066
Ribosomal protein S3Ae	AT3G04840.1	0.001
CLPC homologue 1 (CLPC)	AT5G50920.1	0.0013
Ribosomal protein S11 family protein	AT2G36160.1	0.0014
Cinnamate-4-hydroxylase (ATC4H)	AT2G30490.1	0.0018
Ribosomal protein S13A (RPS13)	AT4G00100.1	0.0018
Ribosomal protein L4/L1 family	AT5G02870.1	0.002
Ribosomal protein S3 family protein	AT2G31610.1	0.0022
Ribosomal protein S5 family protein	AT2G41840.1	0.0023
Ribosomal protein S4 (RPS4A) family protein	AT2G17360.1, AT5G07090.1, AT5G07090.2, AT5G58420.1	0.0029
Plastid transcriptionally active 16 (PTAC16)	AT3G46780.1	0.003
Ribosomal protein L9 (RPL9)	AT3G44890.1	0.003
Ribosomal protein L22p/L17e family protein	AT1G67430.1, AT1G67430.2	0.003
6-phosphogluconate dehydrogenase family protein	AT3G02360.1, AT3G02360.2	0.0032
Translocon at the inner envelope membrane of chloroplasts 110 (TIC110)	AT1G06950.1	0.0038
Structural constituent of ribosome	ATCG00800.1	0.0041
Actin 7 (ACT7)	AT5G09810.1	0.0047
Cobalamin-independent synthase family protein (ATMS1)	AT5G17920.1, AT5G17920.2	0.0049
Glutathione S-transferase phi 8 (GST6)	AT2G47730.1	0.0054
Lipoxygenase 2 (LOX2)	AT3G45140.1	0.0064
Serine transhydroxymethyltransferase 1 (SHM1)	AT4G37930.1	0.0074
Ribosomal protein S15A (RPS15A)	AT1G07770.1, AT1G07770.2, AT5G59850.1	0.0078
ATP synthase subunit beta (ATPB)	ATCG00480.1	0.0083
Actin 8 (ACT8)	AT1G49240.1, AT3G18780.2	0.0089
Ribosomal protein S5/Elongation factor G/III/V family protein (LOS1)	AT1G56070.1	0.011
Light harvesting complex photosystem II (LHCB4.2)	AT3G08940.2	0.011
Ribosomal protein 5B (RPS5B)	AT2G37270.1, AT2G37270.2, AT3G11940.1, AT3G11940.2	0.016
Eukaryotic translation initiation factor (EIF4A-2)	AT1G54270.1, AT1G72730.1, AT3G13920.1, AT3G13920.2, AT3G13920.3	0.016
Phosphoglycerate kinase 1 (PGK1)	AT3G12780.1	0.017
Ribosomal protein L30/L7 family protein	AT2G01250.1	0.017
Ribosomal protein S6e (RPS6B)	AT5G10360.1	0.017
Ribosomal protein L2 (RPL2.1)	ATCG00830.1, ATCG01310.1	0.017
Catalase 2 (CAT2)	AT4G35090.1	0.02
Glyceraldehyde-3-phosphate dehydrogenase C subunit 1 (GAPC)	AT3G04120.1	0.021
Rieske (2Fe-2S) domain-containing protein	AT1G71500.1	0.024
ADP/ATP carrier 1 (AAC1)	AT3G08580.1, AT3G08580.2	0.025
RAB GTPase homolog E1B (RABE1b)	AT4G20360.1	0.025
Mitochondrial substrate carrier family protein	AT5G19760.1	0.029
H(+)-ATPase 2 (AHA2)	AT4G30190.1	0.031
NAD(P)-binding Rossmann-fold superfamily protein	AT4G35250.1	0.033
Formate dehydrogenase (FDH)	AT5G14780.1	0.034
Heat shock cognate protein 70-1 (HSC70)	AT5G02500.1	0.034
Mitochondrial lipoamide dehydrogenase 1 (mtLPD1)	AT1G48030.1, AT1G48030.2	0.035
Cold, circadian rhythm, and RNA binding 2 (GRP7)	AT2G21660.1	0.036
Chloroplast ribosomal protein S4 (RPS1)	ATCG00380.1	0.039
Rubisco activase (RCA)	AT2G39730.1	0.04
Transketolase	AT3G60750.1, AT3G60750.2	0.04
ATPase, F0 complex, subunit A protein (ATPI)	ATCG00150.1	0.046

Relative quantification of total spectrum counts of proteins identified with a confidence level of at least 99% at FDR < 0.1% in each sample containing pAtPNP-A with or without the cross-linker or biotin with the cross-linker (control) from three independent experiments was performed in Scaffold Q+ program. Additional MS data can be found in Supplementary Table 1.

## 2 Experimental Design, Materials, and Methods

### 2.1 Plant materials and growth conditions

Seeds of *Arabidopsis thaliana* (Col-0) were surface-sterilized and vernalized, sown in Jiffy peat pellets (Jiffy Products of America) and grown at 23°C in 16 h of light (200 μmol s^−1^ m^−2^) per day or on Murashige-Skoog agar plates and grown at 23°C in 16 h of light (100 μmol s^−1^ m^−2^) per day for 10 days.

### 2.2 Affinity-based capturing of interactors of AtPNP-A

Peptide containing amino acid sequence of the active region (amino acids: 33 – 66; PYTRSACYGTQRETLVVGVKNNLWQNGRACGRRY) of AtPNP-A protein, followed by RVR linker and biotin tag, termed pAtPNP-A, was synthesized by GenScript (Piscataway, USA) at the purity level > 95% verified with HPLC and the biological activity was verified as described previously [1]. Cross-linking experiments were performed on *A. thaliana* (Col-0) mesophyll cell protoplasts isolated from leaf tissue according to [2]. For each treatment three biological replicates, each of approximately 25 × 10^6^ protoplasts, were prepared and incubated with either 2.5nmol of biologically active pAtPNP-A peptide or biotin (negative control) for 20 min followed by addition of 3,3′-dithiobis[sulfoccinimidylpropionate] (DTSSP) (Thermo Scientific) in approximately 50-fold molar excess compared with the amount of the peptide. To include proteins binding to the peptide without formation of the cross-link, no cross-linking agent was added to one of the samples containing protoplasts incubated with pAtPNP-A. The reactions were incubated in ice for 1 h and terminated with 20mM Tris-HCl buffer, pH 7.5. Protoplasts were lysed in lysis buffer (100mM Tris-HCl, pH 7.2, 5mM DTT) by quick mixing in Vortex mixer. Affinity-based separation of the interactors was performed with the use of streptavidin-coupled Dynabeads M-280 (Invitrogen), previously equilibrated with the lysis buffer, and DynaMag 2 magnet (Invitrogen), according to the manufacturer's recommendations. Non-specific interactors were washed off three times with extraction buffer (50mM Tris-Cl, pH 7.4, 150mM NaCl, 1mM EDTA, 1% (v/v) NP-40, 0.1% (v/v) Triton X-100, 0.1% (v/v) Tween 20, 0.5% (w/v) sodium deoxycholate, 1mM PMSF). Denaturing elution of the bound proteins was performed by 10 min boiling of the beads at 80°C in 1 x SDS sample buffer. Eluted proteins were separated on SDS-PAGE run for 15 min at 100 V, visualized with Coomassie Brilliant Blue, and the bands were excised and subjected to in-gel protein digestion with trypsin [3].

### 2.3 Liquid chromatography tandem mass spectrometry (LC-MS/MS) analysis

Dried peptides were re-suspended in a solution containing 5% (v/v) acetonitrile and 0.1% (v/v) formic acid and analyzed with an LTQ Orbitrap Velos mass spectrometer (Thermo Scientific). Data were recorded with the Xcalibur software version 2.1 (Thermo Scientific) and converted from ‘raw’ to ‘mgf’ with Proteome Discoverer version 1.2.0.208 (Thermo Scientific). All spectra were submitted to Mascot, version 2.4.0, (Matrix Science) and searched against *A. thaliana* in the TAIR database (release 10) containing 35625 sequences, with a precursor mass tolerance of 10 ppm, a fragment ion mass tolerance of ±0.5 Da, and trypsin specificity allowing up to two missed cleavages, carbamidomethyl modification of cysteine specified as a fixed modification, and oxidation of methionine specified as a variable modification. Identified proteins were evaluated and quantified using Scaffold Q+, version 4.10.0, (Proteome Software, Inc.). Peptide identifications were accepted if they could be established at a probability greater than 95.0% by the Peptide Prophet algorithm [4]. Protein identifications were accepted if they could be established at a probability greater than 99.0% and contained at least two identified peptides. Protein probabilities were assigned by the Protein Prophet algorithm [5]. The mass spectrometric proteomics data have been deposited to the ProteomeXchange Consortium via the PRIDE [6] partner repository.

### 2.4 Quantification of identified proteins

Quantification of protein abundance was performed with Scaffold Q+ software. Quantitative profile of the identified proteins was obtained based on total spectrum count (ANOVA test, *P* < 0.05) with peptide and protein false discovery rate (FDR) < 0.1%, where total spectrum counts of proteins identified in samples containing pAtPNP-A with or without cross-linker was greater than total spectrum counts of proteins identified in negative control samples (containing biotin and the cross-linker) from three independent biological replicates.

## Appendix Supplementary materials

Image, application 1

Image, application 2
